# Biomass-Derived Carbon Materials for the Electrode of Metal–Air Batteries

**DOI:** 10.3390/ijms24043713

**Published:** 2023-02-13

**Authors:** Xiaodong Lv, Ming Chen, Hideo Kimura, Wei Du, Xiaoyang Yang

**Affiliations:** School of Environmental and Material Engineering, Yantai University, No. 30 Qingquan Road, Yantai 264005, China

**Keywords:** biomass carbon, air cathode, metal–air batteries

## Abstract

Facing the challenges of energy crisis and global warming, the development of renewable energy has received more and more attention. To offset the discontinuity of renewable energy, such as wind and solar energy, it is urgent to search for an excellent performance energy storage system to match them. Metal–air batteries (typical representative: Li–air battery and Zn–air battery) have broad prospects in the field of energy storage due to their high specific capacity and environmental friendliness. The drawbacks preventing the massive application of metal–air batteries are the poor reaction kinetics and high overpotential during the charging–discharging process, which can be alleviated by the application of an electrochemical catalyst and porous cathode. Biomass, also, as a renewable resource, plays a critical role in the preparation of carbon-based catalysts and porous cathode with excellent performance for metal–air batteries due to the inherent rich heteroatom and pore structure of biomass. In this paper, we have reviewed the latest progress in the creative preparation of porous cathode for the Li–air battery and Zn–air battery from biomass and summarized the effects of various biomass sources precursors on the composition, morphology and structure-activity relationship of cathode. This review will help us understand the relevant applications of biomass carbon in the field of metal–air batteries.

## 1. Introduction

With problems caused by the accelerating deterioration of the environment and the energy crisis, renewable energies such as wind, tide and solar energy have been raised to solve the above problem due to their being widely sourced, environmentally friendly and easily accessible [1,2,3,4]. While most renewable energy is intermittent, which will lead to source waste and further restrict their large-scale application, the development of reliable energy storage devices with high energy and power densities are receiving more and more attention by their combination with renewable energy to realize efficient and sustainable utilization [5,6,7]. As a promising electrochemical energy storage system, metal–air batteries are considered as a suitable energy storage device because of their simple structure, high security, environmental friendliness and especially high energy density [8,9,10]. Among all of the metal–air batteries, the Zn–air battery and Li–air battery have received the most attention due to their huge application potential [11,12,13]. Up until now, Zn–air battery have been successfully used in the field of hearing aids due to their superior secure and excellent energy density. Meanwhile, Li–air battery exhibit the highest theoretical energy density (11,429 Wh kg^−1^) and are regarded as a good successor for next generation energy storage systems [14,15,16]. However, the performances of metal–air batteries are greatly limited by the character of the air cathode, including sluggish oxygen reduction reaction (ORR) and oxygen evolution reaction (OER) kinetics, poor electrical conductivity, and bad nano–micro structure of the cathode, blocked by discharge products, which will lead to high overpotential and poor round-trip energy efficiency [17,18,19].

Although most of the cathodes in metal–air batteries have emphasized the porous structural design and the optimization of ORR and OER catalytic activity to overcome the above problems, the demands for the development of high electrochemical performance electrodes with environmental, inexpensive and abundant properties are still the prerequisite to construct a sustainable energy storage device. Among the various preparation strategies of air electrodes, on the one hand, plenty of efforts have been focused on the design and modulate of morphology and porous structure for the storage of discharge products and efficient mass transfer; on the other hand, no efforts have been spared to improve the interface charge transport and reaction kinetics of the electrode, which are also particularly important [20,21,22]. Considering the above points, it is critical to synthesize efficient, low-cost and porous electrodes for high performance metal–air batteries.

As the second abundant element in the biosphere, carbon (C) is the key component to form life on earth [23,24]. So far, various carbon materials such as Super P [25], graphene [26,27,28,29] and carbon nanotubes [30], which exhibit superior electrical conductivity and controllable structure have been used as air electrodes for metal–air batteries. Unfortunately, the above materials suffer from challenging issues including poor catalytic activities and the lack of suitable structure. Meanwhile, the problems of high energy consumption and pollution as well as cumbersome methods in the preparation of these materials are unavoidable. As a typical and cheap source of carbon, biomass can be converted into carbon materials through the methods of pyrolysis and carbonization. By combining an activation process or utilizing the natural porous structure of biomass, the obtained carbon materials usually possess abundant pore structure and a large specific surface area, which can provide sufficient space and channels for the storage of discharge products and efficient mass transfer. Moreover, the inherent rich heteroatom of biomass can dope into the biochar to further optimize the electrical conductivity and ORR/OER catalyst activities of biochar. For this reason, more and more attention has been paid to introducing porous biomass carbon into metal–air batteries as the air cathode or the substrate catalysts.

A series of papers relative to the utilization of biomass carbon have been reviewed on the fields of environmental remediation, gas adsorption, metal–ion batteries and catalysis, while most of the previous work is focused on the discussion of preparation methods and performance comparison of biomass carbon materials [31,32,33]. From the perspective of the reaction mechanism of metal–air batteries, few reviews have been summarized to explore the influence of different preparation and modification methods of various biomass carbon on its own ORR and OER catalytic activity, and then to conclude its impact on the electrochemical performance of metal–air batteries. In this review, we have extensively researched and discussed the applications and properties of biomass carbon as advanced air electrodes of metal–air, especially Li–air and Zn–air battery. According to the working principle of metal–air batteries, extensive strategies have been used to treat various biomass in order to obtain particular biochar to solve the hard problems faced by the air electrode of metal–air batteries. Furthermore, the reaction mechanism of the obtained biomass carbon in the fields of reducing charge–discharge overpotential and accelerating the deposition and decomposition of discharge products have been discussed. Moreover, the chemical/mechanical stability and structure–electrochemical property relationship of biomass carbon also have been summarized. This review gives a significant exploration and summary of biomass and biomass carbon towards realizing sustainable preparation of high performance air electrodes of metal–air batteries. 

## 2. Raw Materials and Advantage of Biomass Carbon

**Figure 1:**
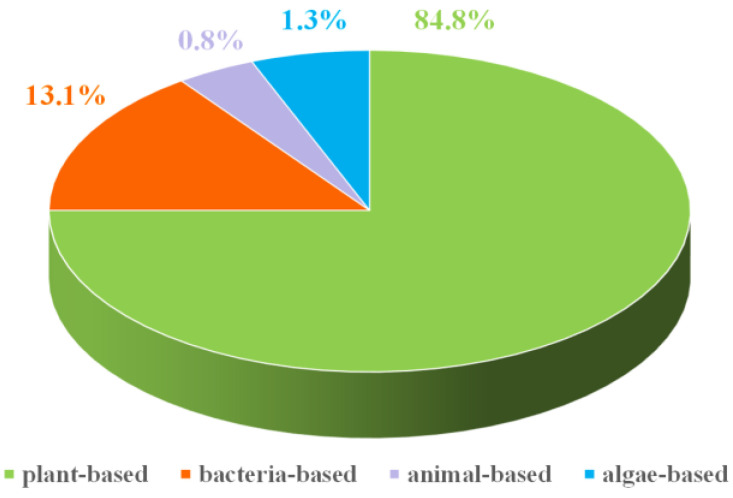
Biomass distribution image on biosphere.

**Figure 2:**
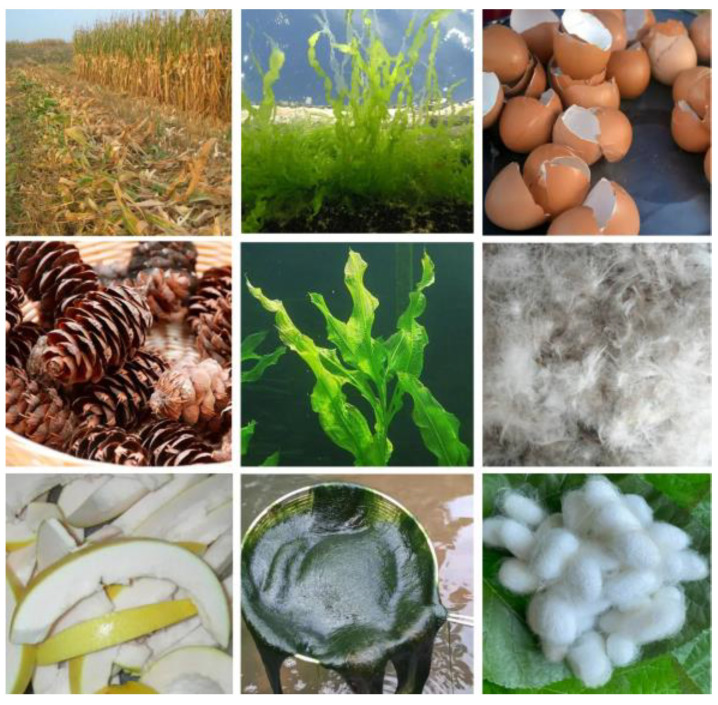
Various biomass produced through plant, animal, algae-based residues.

The biomass of the biosphere is mainly divided into: plant, animal, algae and bacteria-based (Figure 1). The biomass carbon can be mainly extracted from the above four types of biomass. The plant-based biomass is the first rich component in earth, such as forestry (leaves, roots and stems) and agricultural waste (rotten fruit, crops), and exhibits the following characteristics: 1. The inherent sieve tube structure provides a channel for mass transfer; 2. Structural robustness after carbonization; 3. The rich porous structure increases the specific surface area and free space; 4. Desirable conductivity and catalytic performance caused by the heteroatoms contained in biomass [34]. The animal-based biomass can be derived from human household waste, animal components and aquaculture waste, also showing excellent advantages including component advantage (the abundant N, P, S, Fe elements), sustainability, low-cost and high electronic conductivity, etc. The algae and bacteria are important carbon resources from the biosphere; they obtain low-cost, strong reproductive capacity and sustainability. As shown in Figure 2, biomass exhibit a wide range of sources and large quantities. These biomass, being raw materials, are used for the field of energy storage, which plays a crucial role in protecting ecological balance and environmental protection [35]. Moreover, biomass carbon is considered to be carbon neutral because the carbon dioxide released when it is burned is equal to the amount of carbon dioxide absorbed by the plants used to create the biomass. Benefitting from these desirable advantages, biomass as raw materials are widely applied as air cathodes for metal–air batteries, and the air cathode derived from biomass can fully meet the proposed requirements of the air electrode in metal–air batteries. 

## 3. Strategies for Preparing Biochar Materials

The synthetic methods of biomass-based carbon materials serve as key factors in reaching the satisfied physical and chemical properties of the prepared samples. According to the previous literatures, several practical and efficient strategies and methods are widely reported and used for the synthesis of biomass carbon. The most widely used methods of biochar are described in the following section.

### 3.1. Carbonization with Activation 

Activation methods, including physical and chemical activation, serve as the most common method to generate nanoporous structure, and porous carbon can provide abundant free space and increase specific surface area. Under high temperature and oxidizing atmosphere (air, CO_2_, steam), the physical activation process consists of carbonization and activation, whose activation mechanism is described as oxidizing carbon atoms and removing volatile substances in a framework to generate activated carbon with porous structure. Chemical activation is generally divided into alkaline activation and salt activation according to the different activators (KOH, NaOH, K_2_CO_3_, NaHCO_3_, ZnCl_2_). Benefitting from lower activation temperature and higher generation rate of pore, chemical activation is most widely used in the synthesis of biochar, and its activation mechanism is illustrated in the following: (a) First section: activators and its decomposers undergo reduction reaction with carbon atoms in carbon framework, and the carbon substrate obtains the nanoporous structure. (b) Second section: activator is decomposed to generate steam (or CO_2_ and Cl_2_) under high temperature, and the obtained steam being a physical activator participates in physical activation [36,37,38].

### 3.2. Carbonization without Activation

Carbonization without activation, as a common method for the synthesis of activated carbon materials, can avoid the usage of alkali and decrease the cost due to the use of activators being avoided. According the pyrolytic regularity of biomass, the micromorphology of most biomass is reserved in prepared biochar undergoing pyrolysis. Benefitting from the non-introduction of activator, the inherent microstructure of biomass can be preserved by avoiding damage to the alkali [39,40]. Moreover, several types of porous carbon have been synthesized from biomass (corn straw, sugarcane, pomelo peel) with inherent pore structure via carbonization without activation [41].

### 3.3. Hydrothermal Carbonization

Hydrothermal carbonization as an important strategy is widely used to synthesise solid biochar derived from high wetting biomass. In this strategy, biomass are placed in a pressured aqueous medium and converted to biochar at low temperature (100–240 °C). In the process of hydrothermal carbonization, several constituents (cellulose, lignin and hemicellulose, etc.) in biomass are decomposed, undergoing dehydration, polymerization, and aromatization reactions, to synthesize the biochar. In the process of hydrothermal carbonization, the physical and chemical properties of resulting carbon materials are regulated by several factors, such as purser, temperature, liquid medium pH [42,43]. Notably, heteroatoms in self-doped biomass are uniformly distributed in the resulting biochar. Moreover, this strategy is eco-friendly and low-cost compared to the traditional carbonization with ultra-high temperature.

### 3.4. Template Carbonization

Template carbonization is applied to the synthesis of high-performance carbon materials from biomass carbon via a template with excellent structure, and the prepared carbon materials exhibit desirable characteristics, including uniform porous distribution, ultra-high specific surface area and high mass density. During the process of template carbonization, templates, including soft and hard templates, play a crucial role in supporting the framework or providing a carbon source. Notably, several biomass (sugarcane, corn cellulose) with nanoporous structure and low mass density as templates can be synthesized to biochar with high mass density by introducing external carbon sources [44]. For soft templates, such as decomposable polymers, the mesoporous carbon materials are prepared via deleting the template after high temperature carbonization. For a hard template of stable material, its excellent structure can regulate the microscopic morphology (uniform pore distribution, suitable pore size) of resulting biochar. Unfortunately, the cost of preparing biochar is improved by the precise templates [45]. 

## 4. Biomass Carbon-Based Air Cathode for Metal–air Batteries

### 4.1. Outline

**Figure 3:**
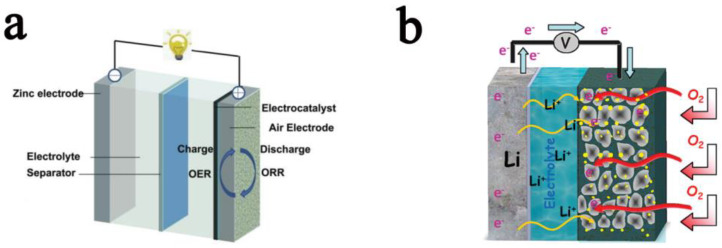
(**a**) Schematic illustration of Zn–air battery. Adapted with permission from ref. [78]. Copyright 2020, American Chemical Society. (**b**) Schematic illustration of Li–air battery. Adapted with permission from ref. [79]. Copyright 2010, American Chemical Society.

For Zn–air battery, the external O_2_ passes through the gas diffusion layer and electrolyte into the cathode, and reduces to OH^-^ under the catalysis of the catalyst [46,47,48,49,50]. For the anode, Zn is oxidized to Zn^2+^ and reacts with the OH^−^ produced by the negative electrode to form ZnO (Figure 3a). The entire chemical reaction can be described by the following electrochemical equation [51,52,53].

Air cathode:

(1) $$O_{2}+2H_{2}O+4{\;e}^{-}\to \;4{\mathrm{OH}}^{-}$$

Anode:

(2) $$\mathrm{Zn}\;\to {\;\mathrm{Zn}}^{2+}+2e^{-}$$

(3) $${\mathrm{Zn}}^{2+}{+4\mathrm{OH}}^{-}\to \mathrm{Zn}{\left(\mathrm{OH}\right)}_{4}^{-2}$$

(4) $$\mathrm{Zn}{\left(\mathrm{OH}\right)}_{4}^{-2}\to \mathrm{ZnO}+H_{2}O+2{\mathrm{OH}}^{-}$$

For a Li–air battery, at the anode, Li metal is oxidized to Li^+^ and migrates from the anode to the cathode to participate in the battery reaction (Figure 3b) [54,55,56,57]. Under the cathode, O_2_ is reduced to O^2-^ and reacts with Li^+^ to form LiO_2_, then follows a series of disproportionation and solvation growth process to generate Li_2_O_2_ storage in the cathode [58,59,60,61,62]. 

Anode:

(5) $$\mathrm{Li}\;\to {\mathrm{Li}}^{+}+{\;e}^{-}$$

Air cathode:

(6) $${\;\mathrm{Li}}^{+}+{\;O}_{2}+e^{-}\;\to {\;\mathrm{LiO}}_{2}$$

(7) $$2{\mathrm{LiO}}_{2}\;\to {\;\mathrm{Li}}_{2}O_{2}+O_{2}$$

(8) $${\mathrm{Li}}^{+}+{\mathrm{LiO}}_{2}+e^{-}\;\to {\mathrm{Li}}_{2}O_{2}$$

We can conclude from the above reaction mechanism that a suitable air electrode for metal–air batteries must have the following characteristics: 1. High electrical conductivity can accelerate the diffuse and transmit of electrons [63,64,65]; 2. Tailored porous structure with a large amount of free space for the storage of discharge products [66,67,68]; 3. Large specific surface area for battery reaction to facilitate mass transfer [69,70]; 4. Efficient ORR/OER electrocatalytic activity to improve reaction kinetics and reduce barriers [71,72,73]. Furthermore, the large surface area and porous structure can avoid the passivation of the electrode caused by the deposition of discharge products even when deeply discharged. Moreover, a large surface area can increase the abundance of active sites and the electrochemical active area for the reaction to take place. On the other hand, the increased surface area also allows for more efficient mass transport of reactants, leading to higher activity and efficiency [26,74]. High electrical conductivity and catalytic activity can improve the energy efficiency and avoid side reactions at high voltage by reducing charge–discharge overpotential [75,76,77].

### 4.2. Biomass Carbon with Heteroatoms Doping 

**Figure 4:**
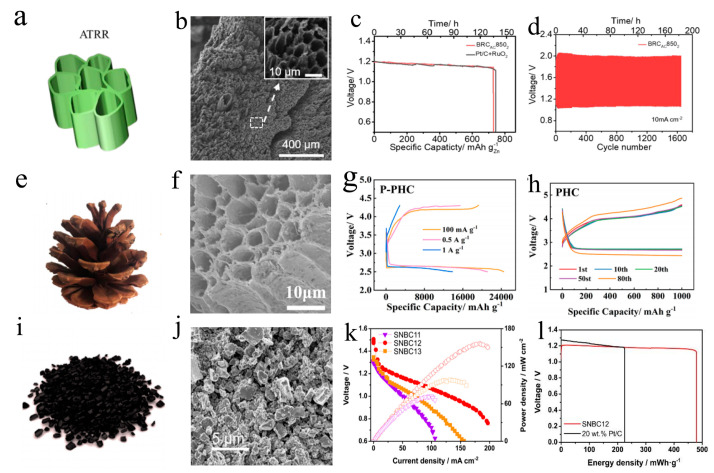
Biochar with heteroatoms, being cathode materials for metal–air batteries; biomass, SEM image of biochars, electrochemical performance curves. (**a**–**d**) Coptis chinensis. Adapted with permission from ref. [90]. Copyright 2019, American Chemical Society. (**e**–**h**) Pinecone. Adapted with permission from ref. [92]. Copyright 2019, American Chemical Society. (**i**–**l**) Bamboo stems. Adapted with permission from ref. [82]. Copyright 2019, American Chemical Society.

Heteroatoms doping is generally regarded as an effective means to improve the electrical conductivity and ORR/OER catalytic activity of materials by regulating the surface and interface properties, and have received more and more attention. Most biomass contain heteroatoms in earth. The percentage of heteroatoms can be impacted by the original composition of biomass, pretreatment conditions, carbonization temperature and carbonization atmosphere. Moreover, the content of heteroatoms have a significant impact on its physical and chemical properties, including the density of the defect, conductivity (the content of graphite nitrogen) and electrochemical catalytic performance (the content of pyridine nitrogen), and so on [80,81,82]. Due to the excellent electron mobility and highly tunable surface properties with the synergistic effect of doped atoms (N, P, B, S) and defects, which are beneficial to the enhancement of electrochemical performance of metal–air batteries. According to the previous report, N atoms can be doped into the carbon lattice in three ways: pyridinic N, pyrrolic N and graphitic N. According to the density functional theory (DFT) calculations, pyridinic N can donate electrons to the carbon lattice and increase the electron density of the carbon lattice, reduce the charge transfer resistance, thus increasing the conductivity of the carbon material. Pyrrolic N can form a conjugated π-bond system with adjacent carbon atoms, thus increasing the electron-accepting capacity of the carbon lattice and improving the adsorption capacity of the carbon material for oxygen [83,84]. This enhanced conductivity and electrochemical activity allows for faster charge transfer, higher energy densities, and rate capabilities. In addition, N doping can also improve the chemical stability of carbon materials, which can reduce the corrosion and is beneficial for the long-term stability of metal–air batteries [85,86]. Liu et al. prepared graphene-like and defect-abundance carbon sheets with N doping (GPNCS) as the air cathode of Zn–air battery from fruits of glossy privet through a hydrothermal-activation-N-doped strategy [87]. The N atoms not only generate abundant defects of biomass carbon, but pyridinic N and graphitic N are able to provide more reaction sites for OER [88]. Owing to the above advantages, the Zn–air battery with GPNCS cathode exhibits excellent electrochemical performance, including a superior cycle stability (1340 cycles at 10 mA cm^−2^) and a positive onset potential (0.92 V). Interestingly, Zhao reported an N-doped porous carbon (NPC) derived from soybean shell via a simple carbonized strategy, and the obtained NPC with a high content of 1.67% N and a large specific surface area of 1036.2 m^2^ g^−1^ after carbonization and 844.0 m^2^ g^−1^ after being sulfonyl functionalized. Benefitting from the compositional and structural advantages, the Zn–air battery with NPC cathode was shown with an OCV of 1.28 V under 111.1 mA cm^−2^ and a positive peak power density of 149.9 mW cm^−2^, reaching a remarkably facilitated electrosorption rate of sodium ions [89]. Especially, the obtained excellent electrochemical performances are attributed to the comprehensive effect of heteroatom doping and porous structure. Jo et al. prepared an N, P co-doped, porous activated carbon (N, P-PAC) derived from tofu as cathode via KOH activation and P doping for a Li–O_2_ battery. Due to component advantages (protein-rich, P-doped) and structural advantage (KOH activation), the Li–O_2_ battery with N, P-PAC cathodes delivered an excellent specific capacity (3700 mAh g^−1^ under 100 mA g^−1^), a lower overpotential as well as a durable cycling life (25 cycles under 1000 mA g^−1^) [37]. As shown in Figure 4a,b, the novel N-doped porous carbon (BRC_AC_850) derived from Acori Tatarinowii Rhizoma is used in the air cathode of a Zn–air battery via a convenient strategy of freeze-drying, N-doping and carbonization. The battery equipped with BRC_AC_850 cathode achieved a durable cycle stability (>1600 times) (Figure 4d) and a desirable specific capacity (>730 mAh g^−1^) (Figure 4c) due to the nitrogenous functional groups and the strong ORR/OER performance of the catalyst [90].

Meanwhile, the P element exhibits characteristics of low electronegativity and a big covalent radius, also playing a critical role in activating adjacent C atoms and enhancing electrocatalytic performance, while promoting the transformation of hybrid carbon into disordered graphite structure and stimulating the production of oxygen-containing functionality for OER [91]. P-doped biomass carbon with porous structure can deliver an excellent catalytic performance of ORR. Jiang et al. used a pyrolysis–hydrothermal strategy to synthesis P-doped pinecone-derived hive-like carbon (P-PHC) with a porous structure (Figure 4e,f), the Li–O_2_ battery with P-PHC cathode exhibited superior performance (Figure 4g,h), including a desirable specific capacity (24,500 mAh g^−1^ at 100 mA g^−1^), an ultra-high working plateau (2.6 V) after 150 cycles, a durable cycling life (205 times at 1000 mAh g^−1^ under 500 mA g^−1^) and excellent rate capacities [92]. Although transition metal particles (TMPs) obtained much interest as promising alternative of noble metal catalysts for ORR, unfortunately, TMPs are easily oxidized due to long-term oxygen exposure during the long-term electrochemical experiment, resulting in the failure of ORR active sites. Importantly, previous studies have shown that the strategy of synthesizing protective layers derived from biomass can effectively prevent the challenging issue of oxidation. Zhang et al. developed an N, B co-doping and purification strategy to synthesize a carbon protective film for Fe_3_C catalyst (D-BNGFe) in a Zn–air battery. During the prepared process, Fe atoms can be used as a catalyst and inhibit the direct combination of B and N atoms to form covalent B-N, which maximizes the synergistic effect of B, N co-doping. The D-BNGFe catalyst lead to a positive onset potential (0.95 V) similar to the Pt/C catalyst, which only exhibits the weak changing of −0.05 V. By analyzing the reaction mechanism, the orphan pair electrons in the dopant activated the adjacent carbon π electrons by conjugation, and the O_2_ molecules were reduced on the adjacent carbon atoms [93]. Notably, the ORR catalyst derived from algae without transition metal still obtain an excellent catalytic performance for the Zn–air battery, which includes greater power density and specific capacity as well as energy density. For example, Zhou et al. selected spirulina as the main carbon source for preparing ORR catalyst (C111-900) in a Zn–air battery owing to low-cost, fast reproduction and high protein content. Benefiting from the two-template strategy of silica and zinc nitrate, the prepared C111-900 exhibit a large specific surface area (1446.0 m^2^ g^−1^) and a desirable mesoporous structure and durability, which is conducive to oxygen adsorption and media migration. The Zn–air battery with C111-900 obtained a satisfactory performance, including high power density (138.5 mW cm^−2^) and specific capacity (766.4 mAh g^−1^) as well as stability exceeding that of commercial Pt/C [94]. Moreover, owing to the excellent interconnected layer structure, the insufficiently burned soot of biomass can also be used as carbon-based supports for catalysts. A microporous carbon nanosheets (F-MNC) with the Fe-N_4_-C structure is derived from corn straw insufficient soot, which is reported by Wang and his colleague. For ORR catalytic activity, the Zn–O_2_ battery with F-MNC cathode obtained a positive E_1/2_ of 0.85 V. Moreover, the Zn–O_2_ battery possessed desirable catalytic activity for OER with an overpotential of 390 mV under 10 mA cm^−2^ and a Tafel slope of 127 mV dec^−1^ [95]. Compared with plant-biomass, animal-derived carbon is significantly less used in metal–air batteries, which is mainly attributed to its poor specific surface area and pore structure. Fortunately, animal-derived carbon with enough heteroatoms owns powerful catalytic performances for ORR/OER. For instance, Wang et al. obtained a strongly active oxygen catalyst derived from silkworm cocoons for flexible and rechargeable Zn–air battery. Although the silk-fiber-derived carbon with poor specific surface area exhibits an unfortunate ORR property. However, the defect-abundance and N-doped carbon (SilkNC/BK) with layered nanometer-thick structure was prepared by carbonizing porous Ketjen black soaked in silk fibroin solution. The obtained SilkNC/BK cathode shows positive onset potential (0.95 V) for ORR and long cycle stability (30 mV loss in half-wave potential after 50,000 cycle), which is caused by three aspects: (a) the electron and mass transfer are facilitated by high conductivity and nanoporous structure; (b) abundant catalytic active sites; (c) the right doping configuration is more important than the total doping content. More remarkably, the Zn–air battery with SilkNC/BK exhibited a specific capacity of 614.7 mAh g^−1^, and good cycle stability including the voltage gap of 1.03 V and the voltaic efficiency of 51.4 V following 100 cycles [96]. 

In order to rebalance the electrocatalytic performance of ORR and low-cost issues, fruit and vegetable peels are conducted as raw material due to high content of sugar, abundant cellulose and large reserves. The application of fruit-peels-derived carbon can fully meet the above two aspects via comprehensive performance of the excellent conductivity, unique structure (high content of microspores) and uniform N-doped distribution. Zhang et al. synthesized a N-doped of porous carbon (CZn_rapid_-400-Melamine-1000) derived from banana peel by a fast catalysis carbonization at low temperature and melamine modification at 1000 °C. The prepared strategy can effectively improve efficiency of production (pre-warm time < 30 min) and achieve ultra-high C conversion efficiency (41.9%). Moreover, the Zn–air battery with CZn_rapid_-400-Melamine-1000 cathode shows a positive ORR activity and durability that CZn_rapid_-400-Melamine-1000 obtained a similar onset potential and reduction current as Pt/C and its CV curves in 1st coincides exactly with the 2000th cycle [97]. Compared with banana peel with N-doped, other biomass carbon obtained multiple heteroatoms (N, S, etc.) also display outstanding ORR activity and robust durability. Ma et al. utilized garlic stems to fabricate N, S co-doped porous carbon (GSC) cathode by the self-activation pyrolysis. The strategy of the self-activation pyrolysis cannot suffer from the high-cost and generating toxic waste of traditional chemical active method. In the special carbonized region, the curved and disordered graphite nano-layers surround amorphous carbon. When the high N, S content of 2.08% and 0.47% were introduced to carbon skeletons, the obtained nano-layers are likely to favor the absorption of oxygen molecules, resulting in improved ORR performance including the max power density of 95 mW cm^−2^ and the excellent durability (the attenuation amplitude of current density recorded at 0.4 V was lower than that of Pt/C after testing at 30,000 s) [81]. The ORR electrochemical catalysts with S, N co-doped carbon (SNBC) are synthesized from bamboo stems (Figure 4i) via pyrolysis and ball-milling method. Based on an environmentally friendly strategy for high-temperature steam activation, S and N elements are introduced into the activated carbon after mixing with thiourea, and the pore structure is changed to micro and mesoporous (Figure 4j). The pore structure of SNBC provides channels for Li^+^ and OH^−^ migration in the electrolyte, and the barrier of migration increases in the small pores, so the pore structure of micro and mesoporous are more suitable for mass transfer. The excellent conductivity of the SNBC is characterized by low charge transfer resistance (97.9 Ω), which is caused by the controlled pore structure increasing the number of active sites. The Zn–air battery with SNBC catalyst exhibited positive ORR catalytic performance, including ultra-high power density (156 mW cm^−2^) and total current density (830 mA cm^−2^). Compared with the Pt/C, the Zn–air battery with SNBC catalyst obtained better electrochemical performance (Figure 4k,l), including higher specific capacity (348 mAh cm^−2^), energy density (340 mWh cm^−2^) and lower ohmic resistance [82]. Liu et al. reported an N-doped porous carbon (NPC) derived from water hyacinth as efficient ORR catalyst in alkaline media. Benefitting from the synergy of nitrogenous functional groups and activation and four-electron reduction pathway, the E_onset_ of NPC is more positive than of Pt/C (+0.95 V), and the limiting current density reached to 3.57 mA cm^−2^, and the NPC exhibited a superior Tafel slope of 103.4 mV dec^−1^ [98].

Moreover, more carbon materials derived from animal-based waste are synthesized as efficient ORR electrocatalysts, such as egg white, blood protein, etc. A nitrogen-doped nanosphere (N-CNSs,) with blood protein as the nitrogen source was synthesized by the pyrolysis method. Thanks to the high nitrogen content of N-CNSs, it can maintain high ORR catalytic performance in an alkaline medium, and can promote the conversion of nitrogen oxide to pyrrole pyridine nitrogen in the precursor, so that the matrix defect density and the exposed edge of the carbon structure are further improved. Nitrogenous functional groups provide more ORR active sites, so the N-CNSs can be applied to a Zn–air battery with alkaline electrolyte [99]. 

Generally, the effective doping of heteroatoms and the porous structure in a biochar-based electrode enables the catalyzing of the ORR/OER of metal–air batteries. Table 1 summarizes the biochar with heteroatoms doping used as electrodes for Zn–air and Li–air battery.

### 4.3. Biomass Carbon Supported with Catalyst

**Figure 5:**
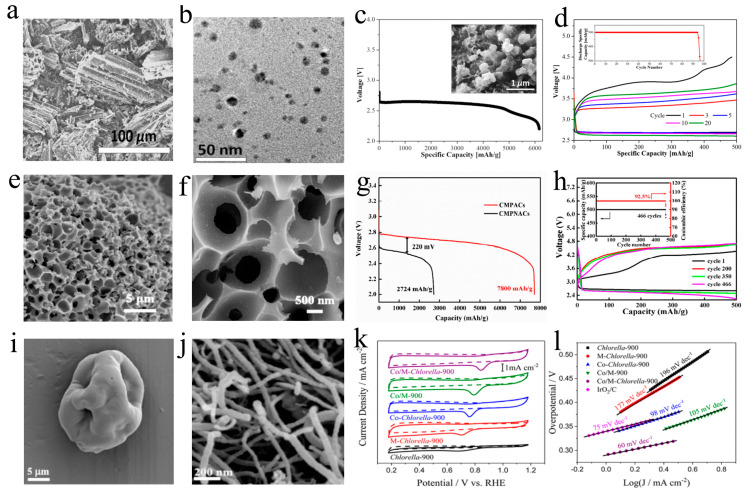
Biochars supported with catalyst being cathode materials for metal–air batteries; SEM image of biochars, electrochemical performance curves. (**a**–**d**) Sugarcane. Adapted with permission from ref. [100]. Copyright 2017, American Chemical Society. (**e**–**h**) Pomelo peel. Adapted with permission from ref. [38]. Copyright 2018, American Chemical Society. (**i**–**l**) Chlorella. Adapted with permission from ref. [113]. Copyright 2017, American Chemical Society.

Although heteroatoms doping can improve the performance of biomass carbon as air electrodes to a certain extent, there is still a long way to go in the pursuit of higher capacity and energy efficiency. The sluggish kinetics of the air electrode are still the main restriction for the practical applications of metal–air batteries. Precious metals such as Ru, Ir, Ag and Pt with excellent ORR/OER activities have been regarded as promising catalysts for metal–air batteries. Meanwhile, the porous structure of biomass carbon is critical for energy transformation and reactant transmission and can provide sufficient storage space for discharge products. As shown in Figure 5a,b, a silver-absorbed ethanol sludge biochar (Ag–ESB) derived from sugarcane is prepared by Yao and colleagues via a carbonization–impregnation strategy. Although Ag–ESB shows a poor specific surface area of 25 m^2^ g^−1^, but the electrochemical reaction and the formation of LiO_2_ can be affected by regulating the particle size of the electrocatalyst. LiO_2_ is treated as one of main discharge products of the Li–O_2_ battery, and its formation can decrease the overpotential for OER. The Li–O_2_ battery obtained Ag–ESB cathodes exhibit desirable capacity (Figure 5c), and stability of the electrolyte after long-term cycling (Figure 5d), resulting from the use of the cathode materials [100]. Due to the rarity and high-cost of precious metals, M (Co, Fe, Mn)-N-C catalysts with good catalytic properties are used as alternatives in the field of metal–air batteries. M–N–C catalysts are known for their high catalytic activity due to the presence of metal–nitrogen–carbon bonds. These bonds provide an environment with a high degree of electron delocalization, which allows the catalyst to more easily promote electron transfer and facilitate faster reaction rates. In general, the presence of M–N–C bonds by metal species could not only improve the electrical conductivity, but also create sufficient localized active sites by adjusting the charge redistribution, which further facilitate the ORR /OER reaction in the metal–air batteries [101,102]. Each metal component in the M–N–C catalyst has its own unique properties, which can have a significant impact on the efficiency of the catalyst reaction. Nickel is known for its ability to promote hydrogenation reactions, while molybdenum and cobalt are often used to increase the rate of oxidation reactions [103,104]. In addition, the metals can also help to reduce the amount of unwanted byproducts and increase the selectivity of the reaction. Moreover, the adsorption energy of the intermediate products on the surface of M–N–C also be regulated to accelerate the efficiency of battery reactions [105,106]. Notably, the metal–N_4_–C on the N-doped porous carbon (NPC) possesses brilliant oxygen reduction performance, about a higher half-wave potential (0.863) than Pt/C (0.856 V). Rong et al. employed natural millet to prepare Co_5.47_N loaded N-doped carbon (CoNMC) through a freeze-drying and pyrolytic method. CoNMC based on Co_5.47_N particles and N-doped carbon substrates is employed by a low-efficient bifunctional electrocatalyst for a Zn–air battery. For the ORR, the CoNMC cathodes show a powerful absorption of hydroxide and gas, revealing Co_5.47_N in CoNMC reduced the barrier of ORR. Owing to the introduction of Co and N atoms, CoNMC exhibited a metallic property, a desirable electrical conductivity and a high defect-concentration. Benefitting from the above factors, the Zn–air battery assembled with CoNMC cathode obtained superior electrochemical performances, including open-circuit voltage (1.51 V), power density (69.5 mW cm^−2^ at 168 mA cm^−2^) and similar cyclic stability (the discharge–discharge overpotential of 1.37 V following 80 cycles) as Pt/C [107]. The N, P and Fe tri-doped nanoporous carbon catalyst (N–P–Fe–C) extracted from corn silk is synthesized by Wan et al. via hydrothermal and two-step carbonization strategy. The ORR catalytic performance of N–P–Fe–C is revealed by the RDE voltammograms, and its onset potential and half-wave potential are more positive than 20 wt% Pt/C. The N–P–Fe–C obtained a better durability than other catalysts. So, the N–P–Fe–C can be one of the best catalyst for ORR. Rightfully, the Zn–air battery with N–P–Fe–C exhibited a specific capacity (625 mAh g^−1^) and a higher voltage (1.38 V under 1 mA cm^−2^, 1.29 V under 10 mA cm^−2^) than the 20 wt% Pt/C [108]. Su et al. fabricated a highly efficient air cathode with ORR activities in flexible Zn–air battery, used a Co and N co-doped method to synthesise the Co-embedded N-doped platanus bark-derived porous carbon catalyst (CNPBPC). The porous structure of platanus bark-derived carbon promoted the diffusion of O_2_. The Co atoms bind to the surrounding N-doped carbon in CNPBPC as a reaction site, and Zn–air battery with CNPBPC obtained superior electrochemical performance, including high open-circuit voltage (1.37 V) and specific discharge capacity (770 mAh g^−1^) [109]. Fe element as an abundant component in biosphere is widely used in electrochemical catalysis. Li et al. designed porous activated and Fe-loading carbon materials (CMPACs and CMPACs-Fe) derived from citrus maxima peel (CMP) as a precursor via a carbonized and KOH-active method. CMPACs-Fe achieved through an activation of the CMP using KOH obtained a high specific surface area of 900 m^2^ g^−1^ and abundant mesopores of 3–10 nm. The excellent structure (Figure 5e,f) can offer a lot of action active sites and three-phase interface for the discharge–charge process, also providing transferred paths for O_2_ and Li^+^, and can reduce concentration polarization and electrochemical polarization. Therefore, the Li–O_2_ battery with CMPACs-Fe achieved a long-term cycle of 466 cycles, a high coulombic efficiency (92.5% after 466 cycles) (Figure 5h) and an ultra-high capacity of 7800 mAh g^−1^ (Figure 5g) [38]. Xu’s group prepared CoN_X_/Zn, N co-doped porous carbon (CoNx/Zn-NC) via self-polymerization of biomass materials and coupling of nitrogen-rich species with metallic ions [110]. The electronic structure of the CoNx/Zn-NC can be adjusted by the introduction of Zn atoms to construct the bimetallic active site (Co-Zn), thereby promoting the adsorption of reaction intermediates and improving the electrocatalytic performance. The Zn–air battery assembled with the CoNx/Zn-NC cathode achieved a specific capacity (718.9 mAh g^−1^ under 15 mA cm^−2^) and energy density (819.5 mW g^−1^). Peach-gum-derived N-doped carbon nanosheets (Co/N-Pg) were prepared by Tian et al. via a hydrothermal-carbonization treatment. For the charge process, the LSV polarization curves showed the positive potential of 1.63 V, and the Tafel slope of Co/N-Pg was lower than other samples (Pt/C, N/Pg, Co/Pg), which revealed the excellent catalytic performance of OER [111]. Meanwhile, researchers reported that Co nanoparticles wrapped in CNT enhanced the ORR/OER catalytic activity, and CNTs can also be employed as a superior skeleton for formation/decomposition of the discharge product, which makes a stable deposition of the discharge product without pore-clogging [112]. Co/M-Chlorella-900, serving as a bifunctional catalyst of ORR/OER, is derived from chlorella via a facile method with N-doped, Co-load and pyrolysis. In the discharge/charge process, the superior electrochemical performance can be attributed to porous and fibrous structure (Figure 5i,j) with the simultaneous increase in ORR (pyridine-N) and OER (graphite-N) active site density. Moreover, the ORR and OER performance are further enhanced by the formation of new active sites through Co nanoparticles wrapped in CNTs, whose unique stereoscopic hollow nest-like structure promotes mass and electron transfer. For instance, in 0.1 M KOH solution, the CV curve apparently shows a positive oxygen reduction peak potential (0.84 V) (Figure 5k), which indicates that the material can be used in the field of aqueous Zn–air battery. The Tafel slope of 60 mV dec^−1^ (Figure 5l) reveals the Co/M-Chlorella-900 catalyst possessed superior OER activity and efficiency [113]. 

Previous studies have also shown that metal oxide nanoparticles such as MnO_x_, Fe_3_O_4_ and RuO_2_ are effective in reducing overpotential during the discharge/charging process [114,115]. In previous surveys, MnO_x_ was one of the most widely applied metal oxides in the field of Li–air battery [116]. The Mn_3_O_4_ nanowires and CNTs composite film with ultrafine RuO_2_ nanoparticles (Mn_3_O_4_/CNTs-RuO_2_ film) is reported by Zhao and his colleagues via a novel prepared strategy of atomic layer deposition. Benefitting from Mn_3_O_4_ as framework with internal interconnection, CNTs are satisfactory conductors, ultrafine RuO_2_ nanoparticles are an effective catalyst for decomposition/formation of the discharge product in a Li–air battery. Owing to the above advantages, Mn_3_O_4_/CNTs-RuO_2_ film can be employed as an integrated cathode in a Li–air battery and exhibited a long-term cycle life of 251 times at 700 mAh g^−1^ under 200 mA g^−1^ and delivered the good discharge specific capacity of 7198 mA h g^−1^ at 100 mA g^−1^ [117]. 

### 4.4. Biomass Carbon with Self-Standing Structure

In general, the activity materials and conductive agents of the traditional spray electrode in metal–air batteries are fixed by binders such as PVDF and PTFE. On the one hand, the use of insulating binders will increase the interface impedance and slow down the electron transfer, on the other hand, the side reaction caused by the decomposition of binders can greatly shorten the battery life [118,119,120]. For this reason, self-standing biomass-derived porous carbon has attracted more attention as binder-free cathode for flexible and non-flexible metal–air batteries with the advantages of superior microchannel structure, desirable mechanical stability and excellent electrical conductivity. Biomass carbon with self-standing structure as current collector and gas diffusion layer as cathode for metal–air batteries, also play a critical role in improve the energy density of metal–air batteries. The catalyst or heteroatoms are uniformly deposited on the surface of the binder-free cathode. Moreover, the desirable structures can be retained after deep discharge. The prepared strategy depends on the conversion of biomass with naturally occurring fibrous and porous structure into uneven cellulose fibers. There are several types of biomass carbon for binder-free cathode, of which the well-known are cereal crop stalks, brinjal, platanus bark, bacterial cellulose and fruit peels.

**Figure 6:**
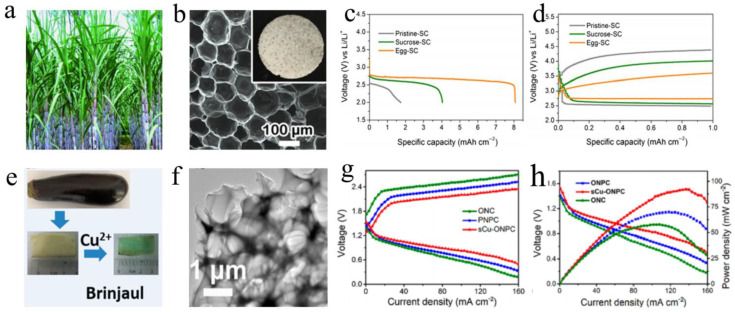
Biochars with self-standing structure being cathode materials for metal–air batteries; SEM image of biochars, electrochemical performance curves. (**a**–**d**) Sugarcane. Adapted with permission from ref. [44]. Copyright 2019, American Chemical Society. (**e**–**h**) Brinjaul. Adapted with permission from ref. [122]. Copyright 2019, American Chemical Society.

Compared with bacterial cellulose of nanofibers, the 3D N-doped carbon nanonet (SCC-N) derived from silkworm cocoons can be used as a carbon-based support for binder-free cathode. Through effective activation, honeycomb pores formed on the surface of carbon fibers with a large specific surface area. The Li–O_2_ battery with SCC-N cathode exhibit ultra-high capacity (1480 mAh g^−1^) and long cycle stability (60 times) due to the excellent structure and morphology of silkworm cocoons [121]. Wang et al. reported a new self-standing air cathode derived from sugarcane (Figure 6a) and egg for a Li–O_2_ battery, due to the egg providing enough O, N, P elements for the carbon matrix derived from sugarcane. Benefiting from the combined effect of double biomass and porous structure (Figure 6b), the Li–O_2_ battery with the egg–SC cathode shows excellent specific capacity (8.07 mAh cm^−2^ under 0.1 mA cm^−2^) (Figure 6c) and delivers ultra-low overpotential (Figure 6d) [44]. For the field of non-aqueous Li–air battery, LiOH being the product of negative reaction can reduce the cycle stability and increase the overpotential of Li–air battery caused by the difficult decomposition of LiOH. Liang and his colleagues obtained a 3D self-standing Co@NC anchored on biochar cathode (Co@NC/PPC) from pomelo peels, and Co@NC/PPC can stimulate the decomposition of LiOH and Li_2_O_2_. Meanwhile, Co@NC/PPC exhibited more excellent characteristics, including high specific surface area (211 m^2^ g^−1^), sufficient micro and mesoporous (1–4 nm) and the hierarchical structure. More importantly, the Li–O_2_ battery equipped with Co@NC/PPC also achieved desirable electrochemical performance, such as a positive ORR E_onset_ (2.96 V), a powerful OER peak (located at 3.2 V), an ultra-high capacity and a relatively long durability (136 cycles with 0.5 mAh cm^−2^ under 0.1 mA cm^−2^). A self-standing biochar (NiFeP/BC) supporting with nickel–iron phosphide nanoparticles was prepared by Liang via a simple strategy of carbonization and one-step phosphating process. Benefitting from the 3D framework with powerful mass transfer and the efficiency catalyst of NiFeP, the Li–O_2_ battery with NiFeP/BC cathode showed a satisfactory specific capacity (>10 mAh g^−1^) and a long-term cycle (90 times) [39].

Biomass carbon with self-standing structure was also widely used in flexible metal–air batteries. Using a porous carbon electrode of O and N coordinated single copper atom active sites anchored, Wang et al. demonstrated a half-wave potential value (0.79 V vs. RHE) and a high-power density of 88.5 mW cm^−2^ at 140 mA cm^−2^ for a flexible Zn–air battery with a prepared N-doped porous carbon (sCu-ONPC) cathode (Figure 6g,h). The sCu-ONPC cathode with Cu anchored exhibits satisfactory mechanical strength and flexibility due to carbon resource of brinjaul (Figure 6e,f) [122]. CNTs-grafted KOH activated carbon (KACC) fabrics derived from medical absorbent cotton is synthesized using a pre-treated, KOH activated and CNTs-grafted method. In field of Li–O_2_ battery, the CNTs on the KACC surface are synthesized to expand the active surface of materials. Moreover, the hierarchical and CNTs-grafted structure of the KACC cathode remains after the stage of deep discharge. The superior durability revealed by the discharge product completely decomposed after recharge. The flexible Li–O_2_ battery with KACC cathodes showed a desirable discharge specific capacity (16.9 mAh cm^−2^ under 0.2 mA cm^−2^) and an excellent cycle stability (150 times). The specific capacity decreased a little from 16.9 mAh cm^−2^ to 11.5 mAh cm^−2^ after the current density increases by 1.5 times [123]. Interestingly, Co and N co-doped carbon (N_x_-wdC-T) derived from wood had received more attention in the Li–O_2_ battery, and N_x_-wdC-T obtained following superior characteristics: 1. Self-supporting structure for binder-free; 2. The formation of Co and N-doped catalyst; 3. Inherently layered pore structure for mass transfer. The Li–O_2_ battery with N_x_-wdC-T cathode reached up to desirable specific capacity (9.44 mAh cm^−2^) and long-term cycle (113 cycles) [124].

In previous studies, the hierarchical and nanoporous structure of binder-free cathode can be entirely preserved after durable cycling. Unfortunately, the mesopores and electrochemical catalyst in the cathode surface are passivated by the sediments which leads the decay of three-phase interface and cycle stability of metal–air batteries. The concept of the renewable cathode is proposed by researchers, which achieves the reuse of cathodes materials by removing deposits on the cathodes. Fortunately, Zhu et al. reported wood-derived carbon (RuO_2_/WD-C) with loading of RuO_2_ nanoparticles as a renewable cathode for the Li–O_2_ battery. The renewable aim of RuO_2_/WD-C is achieved by the efficient removal of sediments on cathodes via a convenient water-cleaning process. More importantly, WD-C obtained excellent conductivity and abundant microchannel for fast mass transition, and the electrochemical performance can be ensured by Ru particles are evenly distributed RuO_2_ particles on carbon matrix. Therefore, the Li–O_2_ battery obtained recycled RuO_2_/WD-C cathodes exhibit voltage curves similar to the initial [40]. 

As a result, the self-standing structure of biomass play a crucial role in improving electrochemical performance, and Table 2 provides the energy density and specific capacity of metal–air batteries (equipped with self-standing biochar electrode).

## 5. Conclusions and Prospects

Biomass, as a widely sourced, sustainable, structurally excellent and heteroatom-rich carbon resource, has been developed as a promising material for the preparation of biochar or heteroatom self-doped carbon materials based entirely on biomass sources. This review introduces and summaries the current state and development of efficient conversion and targeted utilization of biomass to obtain a biomass carbon and biochar-based air electrode for metal–air batteries. Moreover, various strategies of modifying electrode have been proposed by researchers. Firstly, biochar materials can be tailored by activation to obtain a large surface area and porous structure which are favorable to the supply of large free space for the battery reaction and the storage of discharge products, thus improve the capacity of metal–air batteries. Further, the introduction of N, P, S heteroatoms and other metals can provide more active sites and improve the ORR and OER catalytic activity of biochar by tailoring the surface properties to reduce the reaction barrier and realize excellent electrochemical performance. Benefitting from the satisfactory characteristics, including tailored framework, large specific surface area and pore volume, excellent conductivity and catalytic activity, biomass carbon-based air electrodes have achieved satisfactory performance such as longer-term cycle life and higher specific capacity in metal–air batteries. 

It is true that there is still a long way to go to realize the large-scale application of biomass carbon-based materials in metal–air batteries. Firstly, due to the diversity of biomass and the complexity of its composition, the specific chemical components of biomass carbon obtained from biomass after activation and carbonization, including the percentage of heteroatoms, cannot be accurately controlled, which make it difficult to ensure the consistency of its performance as electrode of metal–air batteries. Then, as a universal method of biomass carbon preparation, pyrolytic and carbonization processes often require high temperature and leads to quite an energy consumption, making it difficult to be cost-competitive for large-scale applications. Moreover, owing to the unique environment in which metal–air batteries work in oxygen atmosphere, biomass carbon will inevitably corrode under high voltage and cause a side reaction when it is employed as the cathode of metal–air batteries, thus affecting the battery performance. Finally, in the present, biomass carbon is only used and explored as the cathode in metal–air batteries, which greatly limits its application potential.

To this end, we should still spare no effort to raise the research of biomass carbon in metal–air batteries, including development of new efficient and cheap strategies to realize the precise preparation of biomass carbon with controllable structure and adjustable components; a variety of modification and protection strategies need to be used to improve the stability of biomass carbon to avoid side effects during the charging–discharging process; explore new applications of biomass carbon in metal–air batteries, for example, as the framework of the anode to induce the orderly deposition of Li, Na, K and Zn or as an integrated air diffusion layer to evenly distribute oxygen into the battery. Based on the above, making full use of biomass in metal–air batteries is still an effective way to realize carbon neutrality, waste biomass reuse and the circular economy from biomass to energy.

## Figures and Tables

**Table 1:** Biochars with heteroatoms being electrode for metal–air batteries.

Biomass	Heteroatoms	ORR/OER E_onset_ or Half-Wave Potential (E_1/2_) (V)	Tafel Slope (mV dec^−1^)	Energy Storage Device	Ref.
Glossy privet	N	0.92/1.01		Zn–air	[87]
Coptis	N	1.06/1.68	93	Zn–air	[90]
Pinecone	P	2.7/3.2		Li–air	[92]
Peanut shell	B, N	0.95	59.0	Zn–air	[93]
Spirulina	N	0.96	69.7	Zn–air	[94]
Corn stalk	N	E_1/2_: 0.85 V	108.0	Zn–air	[95]
Silk fiber	N	0.95/1.9	68.0	Zn–air	[96]
Banana peel	N	0.88/1.56		Zn–air	[97]
Garlic stems	N, S	0.97/1.1		Zn–air	[81]
Bamboo	N, S	E_1/2_: 0.81 V		Zn–air	[82]
Water hyacinth	N	0.95	71.1	Zn–air	[98]

**Table 2:** Biochar with self-standing structure being binder-free cathode for metal–air batteries.

Biomass	S_BET_ (m^2^ g^−1^)	Synthesis Method	Energy Storage Device	Specific Capacity/Power Density	Ref.
Egg-sugarcane	166	Infiltration–carbonization	Li–air	8.07 mAh cm^−2^	[44]
Silkworm cocoons	1333	Activation–carbonization	Li–air	1480 mAh g^−1^	[121]
Pomelo peel	211	Impregnation–carbonization	Li–air	14 mAh cm^−2^	[39]
Brinjaul		Auxiliary pyrolysis	Zn–air	88.5 mW cm^−2^	[122]
Cotton		Activation–carbonization	Li–air	16.9 mAh cm^−2^	[123]
Wood		Vapor deposition-carbonization	Li–air	9.44 mAh cm^−2^	[124]
Poplar wood	989	Impregnation–carbonization	Li–air	8.38 mA h cm^−2^	[40]

## Data Availability

Data sharing not applicable. No new data were created or analyzed in this study. Data sharing is not applicable to this article.
